# How Different EEG References Influence Sensor Level Functional Connectivity Graphs

**DOI:** 10.3389/fnins.2017.00368

**Published:** 2017-07-05

**Authors:** Yunzhi Huang, Junpeng Zhang, Yuan Cui, Gang Yang, Ling He, Qi Liu, Guangfu Yin

**Affiliations:** ^1^Department of Biomedical Engineering, College of Materials Science and Engineering, Sichuan UniversityChengdu, China; ^2^School of Electrical Engineering and Information, Sichuan UniversityChengdu, China; ^3^Department of Biomedical Engineering, Chengdu Medical CollegeChengdu, China

**Keywords:** electroencephalograph references, scalp functional connectivity graph, relative error, hamming distance, REST

## Abstract

**Highlights:**

- Hamming Distance is applied to distinguish the difference of functional connectivity network
- The orientations of sources are testified to influence the scalp Functional Connectivity Graph (FCG) from different references significantly
- REST, the reference electrode standardization technique, is proved to have an overall stable and excellent performance in variable situations.

The choice of an electroencephalograph (EEG) reference is a practical issue for the study of brain functional connectivity. To study how EEG reference influence functional connectivity estimation (FCE), this study compares the differences of FCE resulting from the different references such as REST (the reference electrode standardization technique), average reference (AR), linked mastoids (LM), and left mastoid references (LR). Simulations involve two parts. One is based on 300 dipolar pairs, which are located on the superficial cortex with a radial source direction. The other part is based on 20 dipolar pairs. In each pair, the dipoles have various orientation combinations. The relative error (RE) and Hamming distance (HD) between functional connectivity matrices of ideal recordings and that of recordings obtained with different references, are metrics to compare the differences of the scalp functional connectivity graph (FCG) derived from those two kinds of recordings. Lower RE and HD values imply more similarity between the two FCGs. Using the ideal recording (IR) as a standard, the results show that AR, LM and LR perform well only in specific conditions, i.e., AR performs stable when there is no upward component in sources' orientation. LR achieves desirable results when the sources' locations are away from left ear. LM achieves an indistinct difference with IR, i.e., when the distribution of source locations is symmetric along the line linking the two ears. However, REST not only achieves excellent performance for superficial and radial dipolar sources, but also achieves a stable and robust performance with variable source locations and orientations. Benefitting from the stable and robust performance of REST vs. other reference methods, REST might best recover the real FCG of EEG. Thus, REST based FCG may be a good candidate to compare the FCG of EEG based on different references from different labs.

## Introduction

Electroencephalography (EEG) has excellent temporal resolution and is a valuable and cost effective tool for the study of brain functional interactions across a wide range of clinical and research applications (Friston and Frith, 1995; Courchesne and Pierce, 2005; Stam and Reijneveld, 2007; Fogelson et al., 2013; Frantzidis et al., 2014; Van Schependom et al., 2014). It offers a window into the spatiotemporal structure of phase-coupled cortical oscillations that underlie neuronal communication (Tallon-Baudry et al., 1996; Gross et al., 2006; Womelsdorf and Fries, 2006; Fries, 2009; Miller et al., 2009). However, the EEG scalp recording can only provide the potential difference between two points meaning that the use of an appropriate reference is vital (Geselowitz, 1998). This is a problem because no neutral locations exist on the human body (Nunez et al., 1997), and any choice for the reference location inevitably affects the EEG measurements. To minimize this effect, a number of different reference schemes have been proposed including the vertex (Lehmann et al., 1998; Hesse et al., 2004), nose (Andrew and Pfurtscheller, 1996; Essl and Rappelsberger, 1998), unimastoid or ear (Basar et al., 1998; Thatcher et al., 2001), linked mastoids or ears (Gevins and Smith, 2000; Croft et al., 2002), and average reference (i.e., average potential over all EEG electrodes) (Offner, 1950; Nunez et al., 2001). These can provide a relatively neutral reference at least with respect to the signal of interest. Specific laboratories, research fields, or clinical practices have various preferences, and the least biased reference site remains controversial (Nunez and Srinivasan, 2006; Kayser and Tenke, 2010). The lack of a universally accepted reference scheme also represents a major obstacle for cross-study comparability (Kayser and Tenke, 2010).

A neutral potential is required to resolve the problems inherent to using body surface points as a reference. Theoretically, a point at infinity is far from brain sources and has an ideal neutral potential. Therefore, a point at infinity constitutes an ideal reference (infinity reference, IR). Unlike the channel-based methods, such as AR, LR, and LM, Yao (Yao, 2001; Yao et al., 2007) proposed a “reference electrode standardization technique (REST)” to approximately transform EEG data recorded with a scalp point reference to recordings using an infinity reference (IR).

REST has recently been quantitatively validated via simulation studies with assumed neural sources in both a concentric three-sphere head model (Yao, 2001) and a realistic head model (Zhai and Yao, 2004). These studies have shown that data referenced with REST are more consistent with physiology than data referenced using traditional scalp references. This has been shown with a variety of techniques including EEG spectral imaging (Yao, 2017), EEG coherence (Marzetti et al., 2007; Qin et al., 2010), brain evoked potentials (EP) and spatiotemporal analysis (Yao and He, 2003). Previously studies on EEG electrode reference effects have predominantly focused on the power spectra or spatiotemporal analysis; however, there are few reports focusing on EEG reference effects from the perspective of graph theory. This is a significant method to evaluate functional connectivity (FC) networks (Singer and Gray, 1995; De Vico Fallani et al., 2014; Garces et al., 2016). In the realm of FC, Qin (Qin et al., 2010) and Chella (Chella et al., 2016) reported a relatively comprehensive changes on network pattern with different reference schemes. The relative error (RE) (Pereda et al., 2005; Nunez, 2010; Qin et al., 2010) is a metric to evaluate the difference of coherence matrices between each reference scheme and IR. Strictly speaking, instead of describing the FCG similarity (Garces et al., 2016) intuitively, the RE can only detect the global difference between the two matrices. To further evaluate the quantized similarity between FCGs, this study exploited HD as another metric to differentiate the two graphs via the transformed times (Makram Talih, 2005; Medkour et al., 2010; van Wijk et al., 2010; Garces et al., 2016).

One aim of this paper is to get deeper insight into the reference effects on FCGs of EEG with simulated data. Another goal is to determine how the source orientations and locations influence the FCGs from different EEG references. All simulations use an ideal three-shell spherical head model (Yao, 2001, 2017). Four regular references are involved for performance comparison including average reference (AR), the digitally linked mastoid (LM), left mastoid references (LR), and the REST transformation. A coherence matrix (Pereda et al., 2005; Srinivasan et al., 2007; Nunez, 2010) can nicely represent the relationship among EEG channels, and it is utilized to construct a FCG. The reference effects are then evaluated at the matrix level and the graph level. In the matrix level, RE detects the global difference between different references. In the intuitive graph level, HD assesses the difference between connective networks (Makram Talih, 2005; Medkour et al., 2010; van Wijk et al., 2010).

## Methods

### Referencing techniques of EEG

Here, we summarize the most commonly used reference schemes.

#### Reference electrode standardization technique

There are two key points exploited in REST (Yao, 2001, 2017), one is the fact that an approximate neutral reference can be achieved at an infinity point that is far from brain sources, and the other is that activated neuronal sources in the brain are always the same no matter what kind of the reference schemes are utilized (Pascual-Marqui and Lehmann, 1993). Therefore, if we denote *S* as the unknown matrix of the source activities and *G*_*REST*_ as the transfer matrix from these sources to sensors with REST scheme, we have

(1) $$V_{REST}=G_{REST}S$$

where *V*_*REST*_ is the scalp EEG recording with a reference at infinity generated by *S*. Similarly, with the same source activities, the scalp EEG recordings measured with any original reference can be expressed as in

(2) $$V_{REF}=G_{REF}S$$

where *G*_*REF*_ denotes the corresponding transfer matrix of any original reference. Thereby, a linear transformation *T*_*REST*_ can be derived, by combining the above equations, that derives a directly estimate *V*_*REST*_ from *V*_*REF*_ as follows

(3) $$V_{REST}=G_{REST}S=G_{REST}(G_{REF}^{+}V_{REF})=T_{REST}V_{REF}$$

where $G_{REF}^{+}$ denotes the Moore-Penrose generalized inverse and

(4) $$T_{REST}=G_{REST}G_{REF}^{+}$$

From Equation (4), one significant advantage of REST is that EEG inverse problem is not necessary to solve explicitly, that is, the transformation matrix *T*_*REST*_ can be computed without the need to know the actual sources *S*. In fact, only transfer matrices *G*_*REST*_ and *G*_*REF*_ are imperative to construct *T*_*REST*_.

We can calculate *G*_*REST*_ and *G*_*REF*_ based on this ESD rather than on the actual sources because the potential originated by any source can be equivalently produced by a source distribution enclosing the actual sources (Yao, 2003; Yao et al., 2005) and an equivalent source distribution (ESD) on the cortical surface encloses all the possible neural sources. The other main advantage of REST is that, rather than depending on actual EEG data, can only rely on the characteristic of the assumed ESD including the head model, electrode montage, original reference, and spatial geometric. In this study, the ESD is assumed to be a discrete layer of current dipoles forming a closed surface analogous to previous studies (Yao, 2001, 2017; Marzetti et al., 2007; Zappasodi et al., 2014).

#### AR reference, LM reference and LR reference

The reference electrodes should ideally be placed on a presumed “inactive” zone to ensure an arbitrarily “zero level.” The option of the reference depends on the goal of the recording. Frequently, the AR reference, LM reference, and LR reference are adopted. LR uses the right earlobe as a reference, and LM uses the average of both earlobes as a reference. AR, as the name implies, takes the mean of all electrodes as the reference similar to the CZ transformation (vertex) electrode (Lehmann et al., 1998; Hesse et al., 2004). The transfer of data to recordings with reference AR, LR, and LM is easy. A perfect example can be seen in the simulated data derived from an original IR. The results of each reference can be obtained by subtracting the respective reference channel signal from the other channel (Yao, 2017).

### Coherence and network construction

#### Coherence

Coherence is a frequently utilized measure in the analysis of co-operative, synchrony-defined, cortical neuronal assemblies (Pereda et al., 2005; Nunez, 2010). Coherence represents the linear relationship at a specific frequency between two signals *x*(*t*) and *y*(*t*), which can be expressed as:

(5) $$C(f)=\frac{{\left|C_{xy}(f)\right|}^{2}}{C_{xx}(f)C_{xy}(f)}$$

where *C*_*xy*_(*f*) denotes cross-spectral density between *x*(*t*) and *y*(*t*), *C*_*xx*_(*f*) and *C*_*yy*_(*f*) denote the auto-spectral density of *x*(*t*) and *y*(*t*) respectively.

#### Construct the functional connectivity topography

FCG plays an increasingly important role in offering a plausible mechanism for information transfer among neurons (Singer and Gray, 1995; Thatcher et al., 2001; Garces et al., 2016). According to its definition, FCG describes how different brain regions interact with each other while recording signals interact simultaneously (Stephan et al., 2000). A reliable FCG can reproduce the synchronous changes and the interactions between the two brain areas. In this study, a scalp FCG based on EEG is constructed with a coherence matrix, i.e., the coherence among the channels is deemed as the weight of connectivity. To give an efficient representation of network connectivity topography, a connectivity threshold is set to remove weak links between nodes by gradually increasing the connectivity threshold until the degree of each network corresponding to different references reaches four. Therefore, we produce a binary-weighted network.

Affected by the effect of volume conduction (van den Broek et al., 1998), a dense intensity of electrodes may introduce unnecessary or fake links while analyzing the interactions between brain areas. Therefore, 19 nodes are selected from the 129 channels in the EGI montage. These nodes were labeled Ch9, Ch14, Ch20, Ch27, Ch34, Ch36, Ch42, Ch44, Ch62, Ch65, Ch68, Ch73, Ch88, Ch94, Ch96, Ch103, Ch110, Ch116, and Ch121 to approximate the 20 standard electrode locations (Fp1, Fp2, Fz, F3, F4, F7, F8, C3, C4, Cz, T3, T4, T5, T6, Pz, P3, P4, O1, and O2) in the 10–20 system.

### Simulations

#### Simulated source signals

To investigate the robustness and stability of each reference scheme, an EEG connectivity network for each reference was reconstructed by conducting a simulation study. To avoid the effect of volume conduction as much as possible—as well as to better visualize the data—a low-density EEG montage consisting of 19 electrodes from the EGI (Electrical Geodesics, Inc.) 129 system approximating the standard 10–20 system locations was selected.

EEG is mainly used to detect the neuronal activity on the cortex; therefore, rather than deep-level source activity, EEG accurately records the active cortex active from the radial oriented and superficial located dipolar pairs. To clearly confirm the difference between each reference overall, 300 simulated dipole-pair configurations [each consisting of two unit radial dipoles randomly positioned within the upper hemisphere (radius 0.87)] were analyzed. To further determine the feasibility of each EEG reference scheme, 20 dipole pair configurations (each containing two unit radial dipoles with a specific position and 12 different orientations) were analyzed.

**Figure 1:**
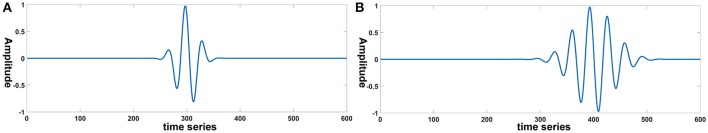
Simulated signal generating two coherent sources where the X-axis represents the sample points, and the Y-axis presents the amplitude of signal. **(A)** The waveform of source 1. **(B)** The waveform of source 2.

Figure 1 shows that two coherent dipolar neural source are generated using a damped Gaussian function, which can be expressed as

(6) $$y(t_{i})=e^{\left(-(2\pi \frac{t_{i}-t_{0}}{\gamma }){\text{​}}^{2}\right)}cos(2\pi f(t_{i}-t_{0})+\alpha )\;i=1,\;2\cdot \cdot \cdot ,k$$

Where, $t_{0}\;=\;100^{*}dt$, *f* = 30*Hz*, γ = 5, $\alpha =\frac{\pi }{4}$ for one dipole in the pair, and $t_{0}=200^{*}dt$, *f* = 30*Hz*, γ = 10, $\alpha =\frac{\pi }{2}$ for the other.

#### Evaluation metrics

##### Relative error for coherence

RE calculates the overall difference between the two matrices, which can be utilized as a holistic approach to evaluate the effectiveness of each reference. Smaller RE values are closer to the reference with IR. Here, RE is calculated as:

(7) $$RE=\frac{\left\|C-C_{*}\right\|}{\left\|C\right\|}$$

where denotes the coefficient matrix of coherence (19^*^19) between channel pairs in specific frequency referenced at infinity, and denotes the coherence coefficient matrix *C*_*AR*_; *C*_*LM*_, *C*_*L*_, *C*_*REST*_ and are calculated with an alternative reference scheme. The matrix norm ||^*^|| is the Frobenius norm defined as

(8) $$\left\|C\right\|=\sqrt{\sum_{i\;=\;1}^{N}\sum_{j\;=\;1}^{N}C_{ij}^{2}}$$

where *N* denotes to the total electrode number, and *C*_*ij*_ refers to the coherence between channel i and channel j.

##### Hamming distance for similarity

Although RE can measure the entire relationship between two coherence matrices from two methods, the accurate relationship of the two elements, which share the same location in two matrices, cannot be measured sometimes due to the effect of square operator. Therefore, another more efficient metric should be considered to measure FCG.

HD is usually used to measure the distance between graphs (Makram Talih, 2005; Medkour et al., 2010; van Wijk et al., 2010). In recent studies on FCG (Singer and Gray, 1995; De Vico Fallani et al., 2014; Garces et al., 2016), HD is introduced to measure the percentage of vector entries that differ. Compared to RE for coherence, the HD can recognize the similarity between two graphs in a more direct way. Given the number of elements of two graphs *G*_*1*_ and *G*_*2*_ with adjacency matrices *N*^(*1*)^ and *N*^(*2*)^ that disagree, HD is defined formally as follows.

(9) $$dist(G_{1},G_{2})=\frac{\sum_{i\;\neq \;j}^{N}\left[N_{ij}^{\left(1\right)}\neq N_{ij}^{\left(2\right)}\right]}{N}$$

The square bracket notation here reflects an indicator function that is equal to one if its argument is true and zero otherwise. The Hamming distance may also be viewed of as the number of addition/deletion operations required to turn the set of edges of into. Smaller HD values results in more similar distances between two FCGs.

##### Comparison between HD and RE

**Figure 2:**
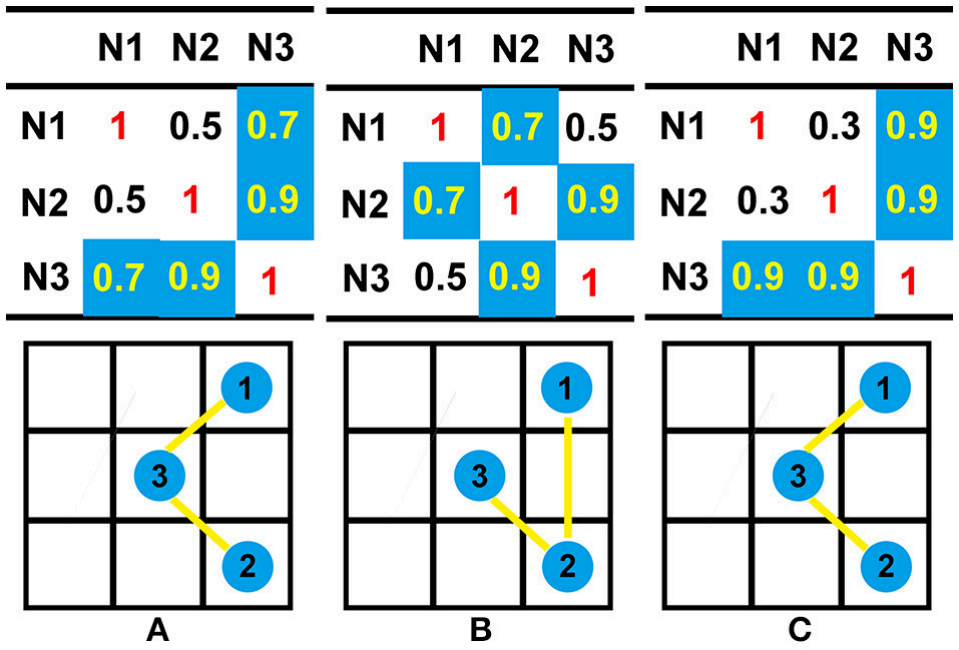
Illustrations of the differences between RE and HD. Here, a coherence matrix of a three-node connection is used as an example. To compare the differences in the matrices from different methods, we set a threshold of 0.55; all connections larger than the threshold are colored with blue, and the connectivity graphs are then calculated from the original coherence matrices. According to Equation (7), the RE between matrix A and B, as well as the RE between matrix A and C, share the same value (0.1172). According to Equation (9), the HD between matrix A and B is 0.4444, while the HD between matrix A and C is zero. The topographies of each matrix are shown in bottom subfigure of **(A)**, **(B)**, or **(C)**; the blue circle denote the nodes, and the yellow lines denote the binary connections between two nodes.

HD is an excellent complement for RE. Assuming that there are three nodes, the 3 × 3 coherence matrices from three different methods are listed in Figure 2. For each node, the coherence for itself is equal to 1. Here, we take matrix A as a standard reference and use RE and HD to evaluate the difference of B and C. According to Equation (7), matrix B and matrix C share the same RE (both are equal to 0.1172). However, B and C are not the same. Especially, in the perspective of connective graph, the two matrices are indeed different from each other. The topographies from matrix B and C—which are combined by the satisfied connections—are quite different when setting the threshold to 0.55 (Figures 2B,C). This difference can then be detected by HD, and the HD for matrices B and C are 0.4444 and 0, respectively. Therefore, despite the similar RE of matrix B and C, matrix C has a smaller value than matrix B. Thus, matrix C is more similar to matrix A than matrix B.

The signal is inevitably mixed with noise in each collection channel. Thus, a good metric should be insensitive to noise. We used 10 groups of data, and each group consists of three matrices—all of which are 5 × 5. In each group, matrix A represents the reference and the other two matrices B and C are used for comparison. B and C can obtain their RE and HD separately by comparison with A. To better investigate the influence of noise on HD and RE, we suppose that B and C in each group have the same overall difference with matrix A but they have different inner connectivity. That is, they share the same RE but different HD. The results of HD and RE are analyzed statistically in different SNR values ranging from 1 to 9. The HD and RE from 10 groups are recorded under specific signal-to-noise (SNR) ratios. Firstly, normal distribution test is exposed on HD and RE to determine whether the two vectors come from normal distribution, Then, the Bartlett test is utilized to determine whether the two vectors own the homogeneity of variance. Finally, if two vectors have the same variance, then a paired test is then exploited to conduct a test decision whether two vectors share the same equal mean. The Bartlett test results of HD and RE illustrate that by adding noise with specific SNR, the intragroup HD and RE can maintain the normal distributions with the same variance (p-value > 0.05). Paired-test results show that; intragroup HD can hold the stability in distinguishing matrices with various SNRs (p-value < 0.05), while intragroup RE cannot recognize the difference between matrices even in high SNRs (p-value > 0.05). The Appendix discusses in more detail the effects of HD and RE on evaluating the similarities between two FCGs (Supplementary Material).

### Configurations of simulations

#### Simulation 1: reference effects on two fixed dipoles

A general case is shown in two fixed dipoles, and the configuration of the corresponding sources are set as follows: one dipolar is set in with orientation vector, and the other is set inwith the orientation vector. Both the simulated source signal is in the form of a damped Gaussian without any noise (see Figure 1).

#### Simulation 2: reference effects on superficial and radial dipoles

To explore the influence of orientation on difference references, various orientation combinations were used for the simulations. Source orientations in the human brain are dynamic, and thus a good reference scheme should be insensitive to changes in source orientations. To investigate the stability and robustness of each reference scheme, different orientations that contain almost all of the possible combinations of basic orientation components of sources should be applied to each simulated dipolar pair.

Inspired by Qin et al. (2010), the performance of each reference scheme with 300 random distributed dipolar pairs was investigated. However, in their work, the factor of source orientations was discussed only in passing. Their results from deep sources have not yet been clearly detailed. Therefore, we further explored the source direction in this study. Twenty dipolar pairs were considered, and each pair contained 12 orientations.

In this simulation, we used 20 dipolar pairs with a large scale of variations on orientations and locations. While the variation between each dipolar pair is distinct, the distributions cover almost the entire possible active area in the cortex. These are primarily located in four situations including bottom-up, left, right, central, and left-right (Table 1).

**Table 1:** Illustrative Maps of Distribution of the 20 Dipolar Pairs [colorful solid points (green and yellow) denote the simulated sources].

**Source distribution types**	**Illustrative maps**
Bottom-Up	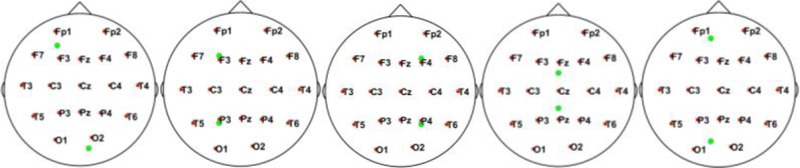
Left	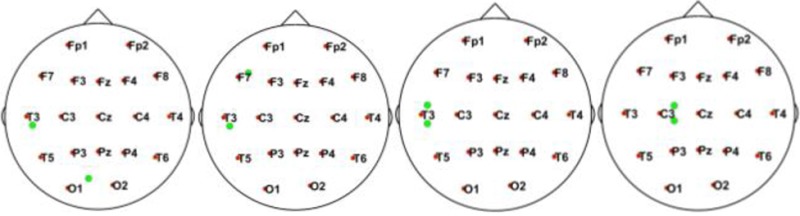
Central	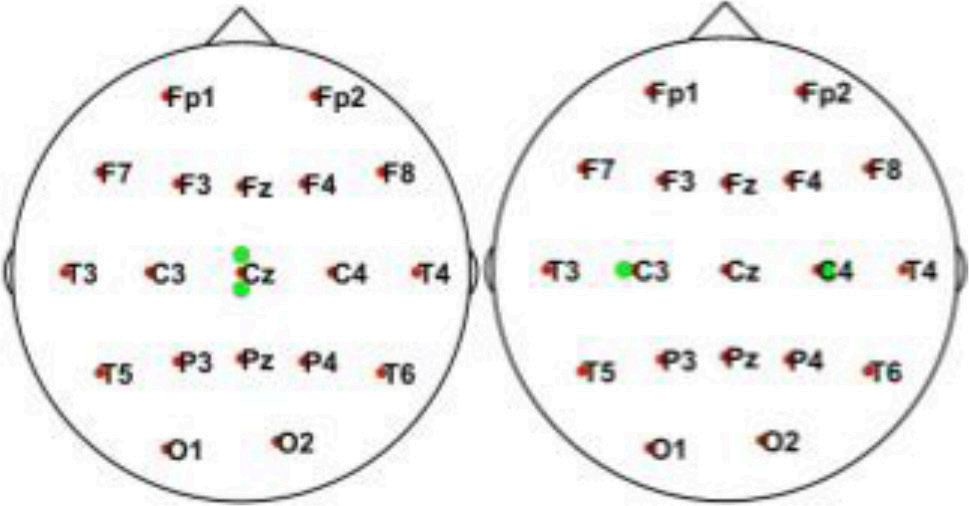
Right	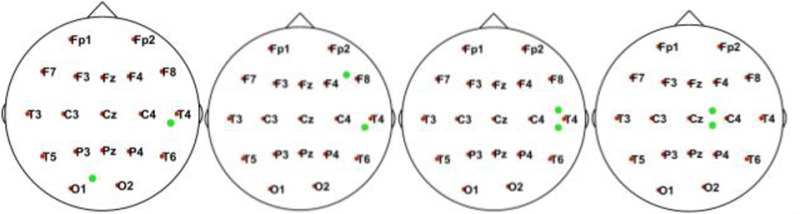
Left-right	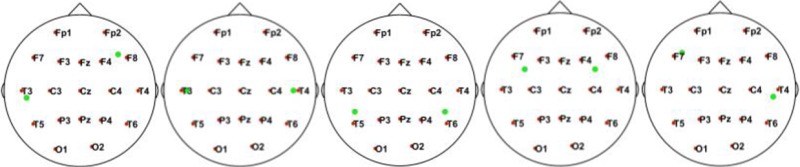

*Source is projected on the z = 0 plane*.

To evaluate the stability of the different reference schemes in all possible directions, 12 orientation combinations were applied to each dipolar pair, respectively. The vector of each orientation is represented in the three unit components, i.e., the unit along the X-axis, Y-axis, and Z-axis. The combinations are listed in Table 2.

**Table 2:** Orientation combinations used in each dipolar pair (applicable for 20 dipolar pairs with complicated orientations).

**No. of orientation combinations**	**Source 1**	**Source 2**
1	(1,0,0)	(1,0,0)
2	(1,0,0)	(0,1,0)
3	(1,0,0)	(0,0,1)
4	(0,1,0)	(0,1,0)
5	(0,1,0)	(0,0,1)
6	(0,0,1)	(0,0,1)
7	(1,0,0)	(−1,0,0)
8	(1,0,0)	(0, −1,0)
9	(0,1,0)	(0, −1,0)
10	(1,0,1)	(1,0,1)
11	(1,0,1)	(0,1,1)
12	(1,0,1)	(−1,0,1)

All the electrodes and the simulated dipolar pairs were projected into the central transverse section in simulations. This better reveals the relative temporal relationship of each electrode in one plane. To give a better representation of the network connectivity topography, a connectivity threshold was used to remove weak links between nodes. The threshold was increased by decreasing the network degree (mean number of links per node across the network) until the degree of each network reached two.

## Results

### Simulation 1: reference effects on positions fixed two dipoles

**Figure 3:**
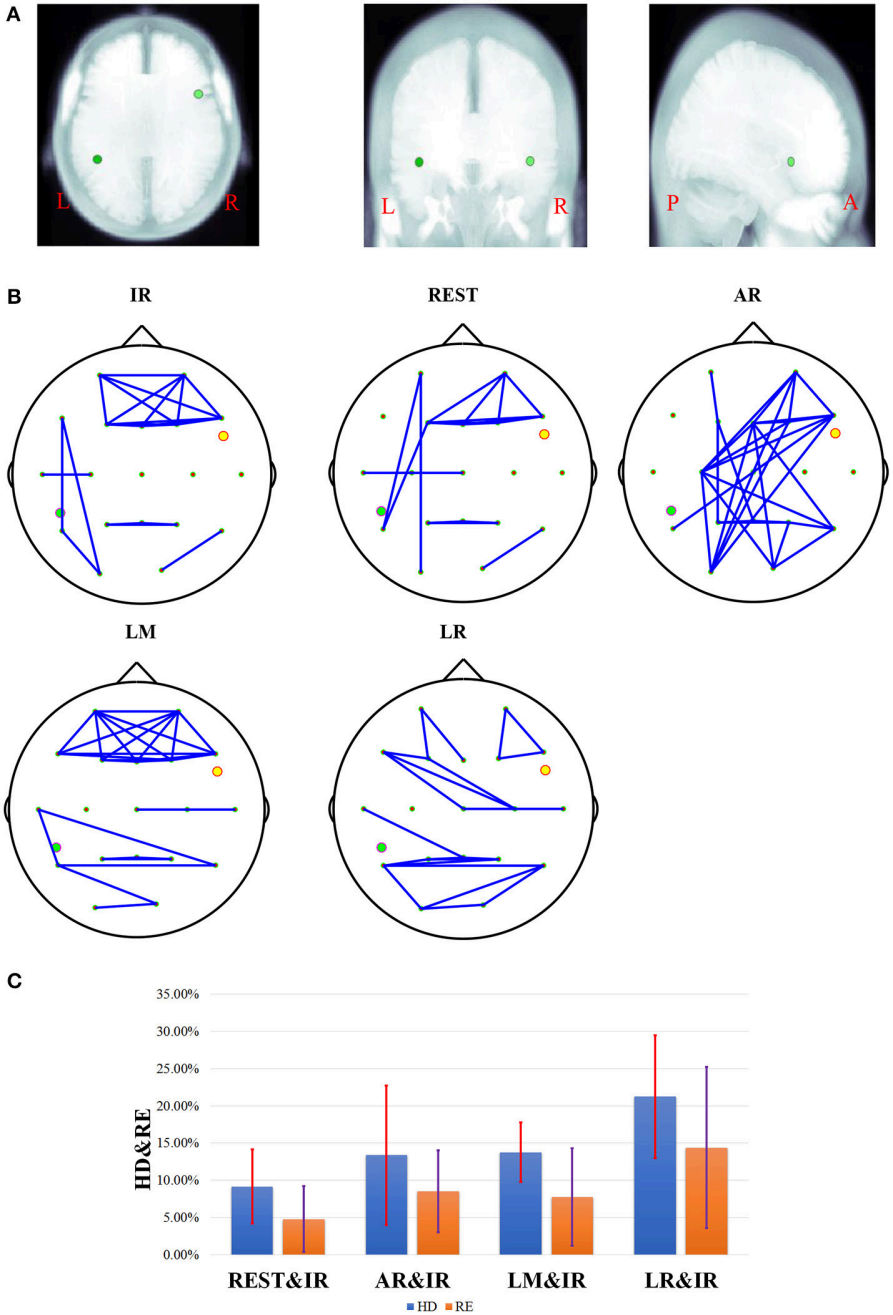
The results of one fixed dipolar pair, one dipolar is set in [−0.6, −0.3, −0.4] with orientation vector. The other is set in [0.6, 0.3, 0.4] with the orientation vector (0,1,1). Here, **(A)** is the location of the dipolar pair overlaid on MRI structure images. The MRI structure comes from Brainstorm anatomy template ICBM512. From left to right, this shows the location of the two simulated sources under the view of axial, coronal, and sagittal respectively. Here, L denotes the left, R denotes the right, P denotes the posterior, and A denotes the anterior. In **(B)**, the network connectivity topography is a dipolar pair with different references. **(C)** Results of different references on one fixed dipolar pair involving 12 orientations. The blue bar represents the results of HD, and the orange bar represents the results of RE. The red and violet segments denote errors sources from different references in HD from 12 different orientations and RE results from 12 different orientations, respectively.

To illuminate the source location vividly, a standard three-view MRI structure was used from an anatomy template ICBM512 in Brainstorm. Sources location in *Simulation* 2 are shown in Figure 3A, and the corresponding FCGs are shown in Figure 3B. The RE and HD statistics are shown in Figure 3C. Taking the FCG of IR as a standard, REST obviously has the most similarity with IR at the first sight, and AR FCG is the most disordered (Figure 3B). Here, HD is used to evaluate the graph similarity, and the quantized performance of each method is *HD*_*REST*_ = 7.2%, *HD*_*AR*_ = 16.96%, *HD*_*LM*_ = 9.94%, *HD*_*LR*_ = 14.62%. This agrees with the exhibited connectivity topographies.

In no-noise simulation, RE is an efficient metric to illustrate the accuracy of different schemes quantitatively. However, the persuasiveness of RE in FCG is not that intuitive. HD is a complementary metric, and it can measure the distance between each reference schemes and IR with respect to graph similarity. Theoretically, for each reference, smaller HD and RE values result in values that are more similar to the IR. This further improves the method. On the fixed location, the results of different combinations of orientation are shown Figure 3C, and REST is closer to zero than the other three reference schemes from the perspective of average HD and RE. While the standard REST is higher than that of LM, the entire range of REST is closer to zero than LM.

### Simulation 2: reference effects on superficial and radial dipoles

Theoretically, if the active source is located on the superficial cortex and the source direction is radial, then EEG can detect and recover active signals very well. Therefore, a good EEG reference must have an excellent reflection of the source activation—especially the superficial and radial cortex source. The RE and HD metrics are utilized to evaluate the difference for each reference from the perspective of coherence matrix and the similarity of FCG.

**Figure 4:**
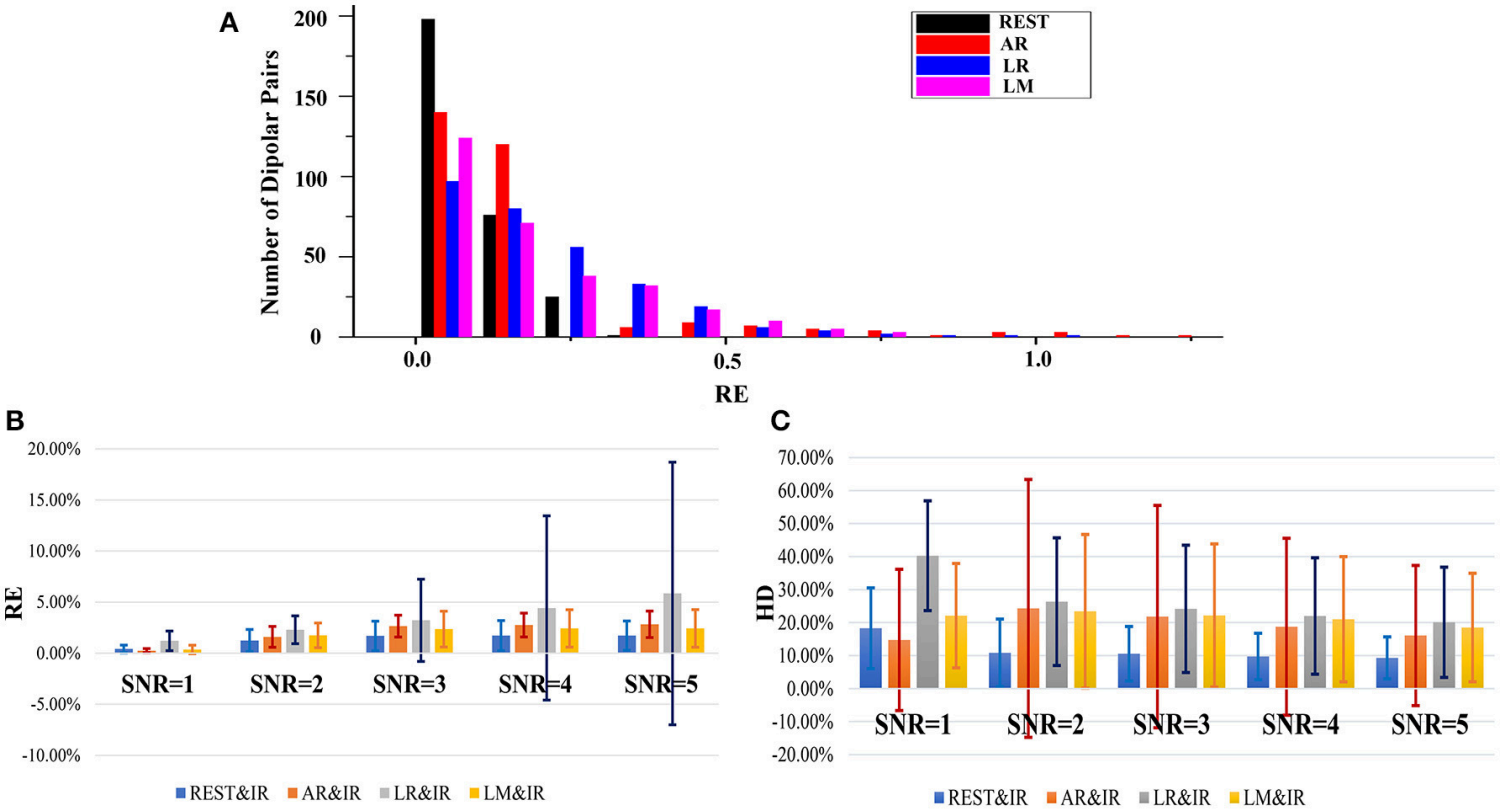
Statistical results of RE and HD based on 300 radial and superficial dipoles. **(A)** Relative error histogram of each reference where the SNR of each dipolar pair is 5. The black bars denote the number of the results from REST in different RE values, and the red, blue and magenta bars represent the results of AR, LR and LM, respectively. **(B)** The results of RE between each reference and IR on 300 dipolar pairs. The results are shown for different SNR conditions where the blue bars denote the RE results for different SNRs. The orange, gray, and yellow represent the RE results of AR, LR, and LM, respectively. **(C)** HD results between each reference and IR based on 300 dipolar pairs. The results are seen for different SNR conditions where the blue bars denote the RE results for different SNRs, and the orange, gray, yellow represent the RE results of AR, LR, and LM, respectively.

The histogram can reflect the distribution of results at different levels. Figure 4A shows RE histograms of each reference in a noisy situation (SNR of 5). There are 300 dipoles with REs between REST and IR, 200 diploes are nearly zero, and almost 75 dipoles are around 0.1. However, for REs between AR and IR, only ~125 dipoles are nearly zero. A comparative number of dipoles are around 0.1. The remaining dipoles are distributed across a relatively large scale of variation. The LR situation shows a worse result—fewer than 100 dipoles are obtained from the nearly zero RE. There are fewer than 200 dipoles with RE values of 0.1. As for LM, the distribution scale is larger than REST, and the number of RE that is less than 0.1. These only occupy half of the total.

During EEG measurements, the electronic disturbance from noise must be considered. A good reference should have a stable performance at different noise levels. Figures 4B,C shows that when the noise is difficult to distinguish from signal, then SNR equals 1. Here, the EEG measurements at all references lose efficacy. However, when SNR is greater than 1, REST is much better. Clearly, the averages of REST RE in different SNRs (≥2) from 300 dipoles are all around 0.1. The REST HD are all below 0.025. The RE and HD of other references are almost twice as high in terms of average and variation. The REST RE and REST HD have relatively smaller values and vary on a smaller and more stable scale. AR in particular varies more sharply than other references in different SNRs.

### Simulation 3: reference effects on 20 dipoles with various orientation combinations

**Figure 5:**
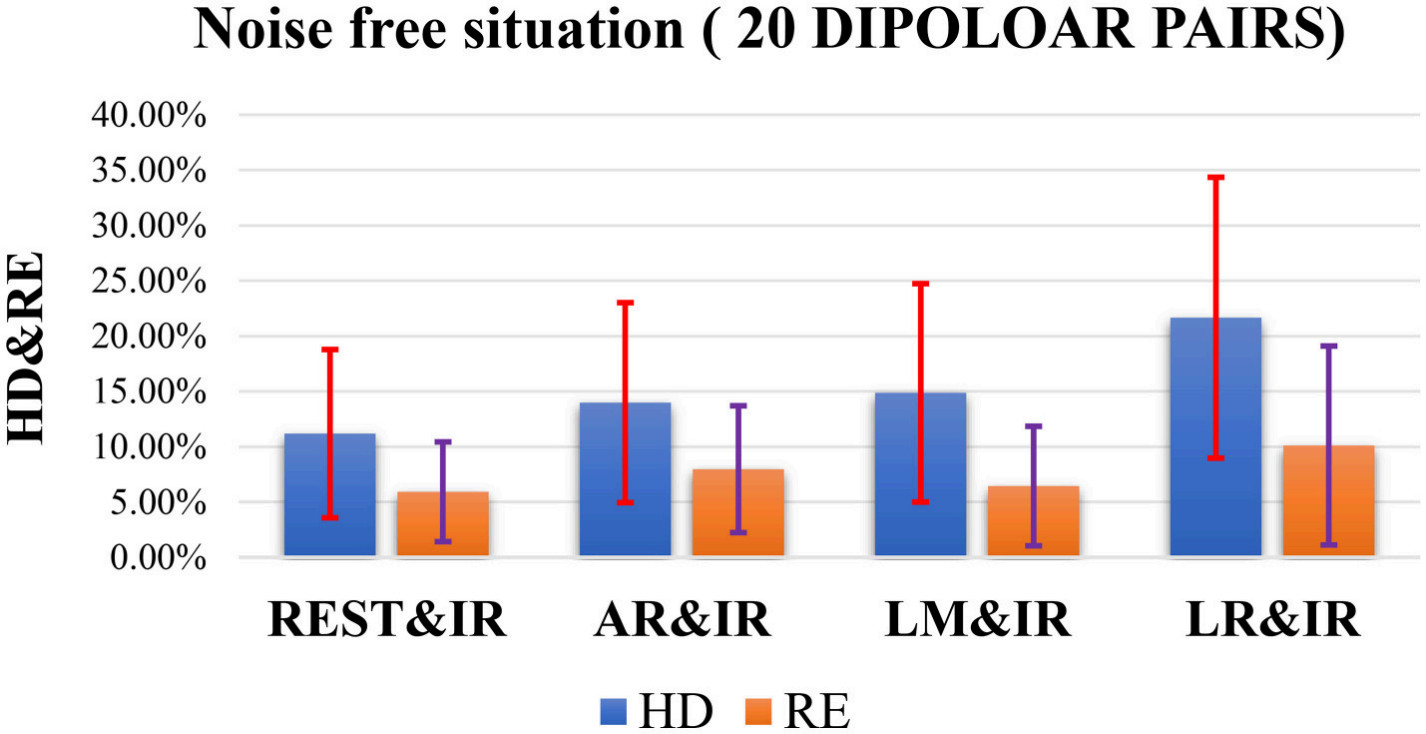
Results of HD and RE on 20 dipolar pairs in a noise free situation. The blue bar represents the results of HD, and the orange bar represents the results of RE.

Figure 5 shows the overall statistical results of HD and RE. These are consistent, i.e., RE tends to be similar to HD at each reference. While these are affected by the distributed form of sources, REST also shows a better performance than the other methods. Statistically, REST has the smallest average RE and HD as well as the smallest fluctuation (Table 3). The HD and RE variations of REST are both about 5%; other references are much greater. Thus, REST seems to be a better reference choice. Figure 5 shows that LR is obviously the worst choice. It has a high average and variance; the performance of AR and LM is moderate.

**Table 3:** Statistical results of HD and RE on each reference in a noise free situation (20 dipolar pairs; each pair with 12 orientations).

	**Hamming distance (%)**	**Relative error (%)**
REST	11.18 ± 7.60	5.93 ± 4.51
AR	13.98 ± 9.04	7.97 ± 5.73
LM	14.87 ± 9.87	6.45 ± 5.4
LR	21.66 ± 12.69	10.11 ± 8.99

**Figure 6:**
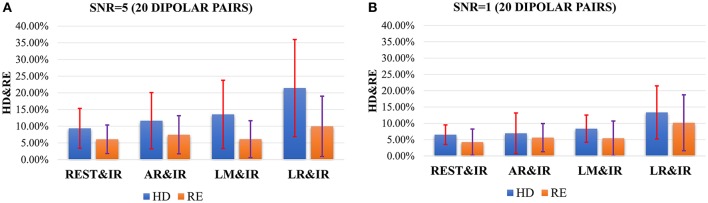
HD and RE results of 4 references with different SNRs. The blue bar represents the results of HD, and the orange bar represents the results of RE. **(A)** Results in the case of SNR = 5; **(B)** Results in the case of SNR = 1.

The results in Figure 5 do not consider noise. However, scalp electrodes always contain real EEG and noise. Thus, to verify the robustness of the different methods in a real situation, we simulated the signals with different SNRs by adding random Gaussian noise considering both poor and good situations. Once the location of each dipolar pair is determined, random Gaussian noise is added to the ideal source signal. This is repeated 100 times. Figure 6 shows both high SNR (SNR = 5) and low SNR (SNR = 1) vs. other methods. The average and standard deviation of HD and RE from REST is the minimum. Thus, in a noisy situation, REST achieves relatively higher robustness.

In ideal (no-noise) situations, the orientation of the dipolar pair significantly affects the performance, in addition to the positional influence on each method. To investigate the stability of each reference scheme with these inevitable variable factors, the results in each orientation are considered separately by exploiting HD as a direct metric.

**Figure 7:**
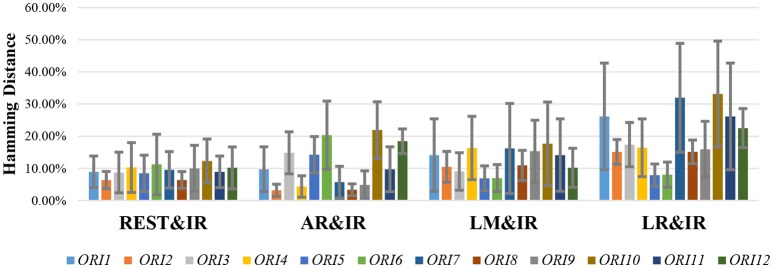
HD results of different references on 20 dipolar pairs. Each pair contains 12 orientations. Color bars represent the average value of 20 dipolar pairs on each orientation, respectively, and the error bars represent the standard deviation of 20 dipolar pairs on each orientation.

In fact, the real orientation of the dipolar pair is usually complicated; therefore, a good zero-reference scheme should be promising with a stable tolerance in many possible orientations. Figure 7 shows that even though REST may not always have the best performance, it is the most stable. AR, LM, and LR have good performance in limited. According to Figure 3, REST should achieve excellent performance when the source active is superficial and radial, but it is affected by the deeper simulated source (Figure 7). The REST has undesirable performance in ORI*6* (orientations of two source that are both radial). Although REST has poor performance in ORI*6*, REST is better in AR.

AR operates better than REST under certain orientations, but it performs worse in many orientations like *ORI3, ORI5, ORI6, ORI10*, and *ORI12* that contain the upward component in dipolar pair. Since AR fluctuates largely with the change of orientation, AR maybe not a good choice for zero-reference. LR and LM are limited by their own strategy and are largely affected by the source position. In the simulated 20 dipolar pairs, the amount of symmetry distribution is larger than the asymmetry distribution. Therefore, LM performs better than LR.

## Discussion

EEG results from different reference sometimes vary widely. They are influenced by the inevitable reference issue and are limited by the principle of EEG. Here, we studied EEG reference effects on FCG with AR, LR, LM, and REST. Each reference has specific zero-reference schemes. The LR systematic decreases the EEG amplitude in the electrodes, and these are closer to the reference side. Although the LM reference makes use of “linked” earlobes, asymmetry from LR reference is avoided, but this distorts the EEG mapping because the electric current flows inside the linking wire. This affects the intracranial currents that form the EEG potentials. AR avoids asymmetry from LR or LM. However, vs. REST, the AR reference needs several strict conditions to gain zero integral assumptions: (1) sufficiently dense electrodes, (2) complete electrode coverage (sampling both the upper and lower part of head), and (3) the head must be spherical (Nunez and Srinivasan, 2006; Yao et al., 2007). Such ideal conditions are rarely realized. in contrast to REST, the AR reference, LM reference, and LR reference are all theoretically based on the channel transformation. The unexpected activity would be largely induced to the referenced recordings because the specific channels are not electrically active. Therefore, channel-based references are not that recommended (Yao and He, 2003).

It must be acknowledged that RE (Pereda et al., 2005; Nunez, 2010; Qin et al., 2010) can well reflect the overall difference between the two matrices and has its irreplaceable superiority on measuring the difference between graphs, thus RE has been widely adopted to evaluate the difference between coherence matrices from EEG references. However, evaluations on EEG references which only depend on RE are not sufficient. A perfect example can be found that, if two graphs share the same whole difference but their inner networks are changed, RE cannot detect the difference between the two graphs. To complete RE, HD (Makram Talih, 2005; Medkour et al., 2010; van Wijk et al., 2010) is induced as a new metric, which can well evaluate the difference in topographies. Derived from graph theory, HD can effectively detect the edge changes in networks. Even though, unlike RE, HD cannot measure the entire difference of weights, it is relative intuitive and objective to detect alterations in FCG. Thus, as a complementary, HD contributes to helping complete the detection of RE by measuring the alterations in networks. For example, in *Simulation* 1, the difference of RE between LM and REST is too subtle to detect. But by combing the two metrics, we can evaluate the similarity of graphs more precisely, so that we can better study reference effects on FCG. The two metrics have their unique superiority, and they can make their respective advantages complementary to each other. Therefore, we should choose the appropriate evaluate metrics according to the practical issues.

The results of RE and HD validates that REST performs well in terms of both stability and robustness. REST works because it grasps the essence of the zero-reference. AR can average the signal and noise from each electrode; thus, it achieves good performance when the orientation of the source is along with the axial plane or under noisy situations. However, once there is an upward component in the source orientation, the baseline of AR is abnormally high. Thus, thus performance of AR is unsatisfactory. Although LM and L are insensitive to the orientation of sources, the results depend significantly on the distribution of sources. LM would achieve a stable performance especially for of bilateral symmetry of sources. LR requires rigorous conditions to achieve good results, i.e., LR is close to IR only when the location of the source is far from the left ear. We conclude that REST can achieve stable performance under diverse situations, while AR, LM, and LR can achieve satisfactory results only in a few situations.

## Conclusions

In this study, we investigated how different reference choices influence FCG using simulated EEG data with various SNR values that were generated from different source combinations. The simulation shows that reference choices have a significant effect on coherence—a measure that indicates synchronization and interaction. As a result, the FCGs also differ across reference schemes. The RE or HD between REST and IR had the smallest values relative to AR, LM, and LR references as well as IR. This means that REST reconstructs FCG better than IR. Moreover, the results revealed that REST could perform stably even when the sources vary on orientations compared to other reference schemes. These findings indicate that the choice of reference plays a crucial role in functional network studies in the brain. It is critical to consider this thoughtfully. REST is the recommended reference technique for objective comparisons as well as cross-laboratory studies and clinical practice.

## Author contributions

YH: Simulate the designed experiments and evaluate the results, Write the whole manuscript. JZ and QL: Design the whole experiments and Revise the entire framework of the manuscript. YC, LH, GaY, and GuY takes part in analyzing the logic and checking the grammar error of the manuscript.

### Conflict of interest statement

The authors declare that the research was conducted in the absence of any commercial or financial relationships that could be construed as a potential conflict of interest.
