# Supplementary material for: How Different EEG References Influence Sensor Level Functional Connectivity Graphs
Source: Front Neurosci. 2017 Jul 5;11:368. doi: 10.3389/fnins.2017.00368 (PMC5496954; doi:10.3389/fnins.2017.00368)
Supplement: Supplementary file 1 [file Presentation1.PDF]

Appendix

Discussion on HD and RE

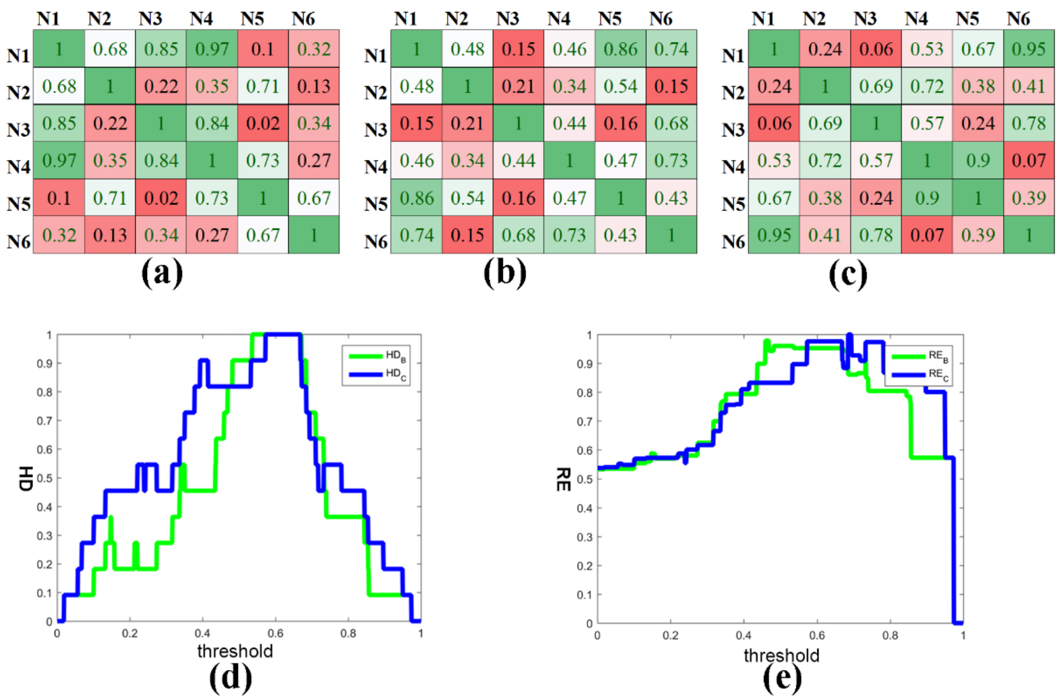

Figure 1 Simple examples to compare HD with RE. a) Random generated matrix A (6*6), and it is utilized as the reference matrix. b) Random generated matrix B (6*6). c) Random generated matrix C (6*6). Different values are mapped into different colors. d) Results of HD. The green line represents the HD values between B and A varying with different thresholds. The blue line represents the HD values between C and A varying with different thresholds. e) Results of RE. The green line represents the RE values between B and A varying with different thresholds. The blue line represents the RE values between C and A varying with different thresholds.

Figure 1 exhibits a simple example with three random generated matrices. Assume that there are six nodes in each graph, just as it is shown in Figure1(a), (b) and (c), all the matrices are in the size of 6*6, and the value of each element denote the connective weight between two nodes. To better illustrate the difference within matrix, values that locate in different scales are colored different. Matrix A is taken as the standard, and the other two matrices B and C are thought as results from two different approaches. Figure 1(d) and (e) show the HD and RE results varying with different thresholds. By comparing with A, green lines in Figure1(d) and (e) represent the HD and RE results of matrix B respectively. The blue lines represent that of matrix C.

From Figure 1(d) and 1 (e), when threshold is set in the range from 0 to approximate 0.5, HD can have distinguishable results between B and C, while RE can hardly tell which matrix is closer to the standard. When threshold is larger than 0.5, HD and RE share a similar performance.

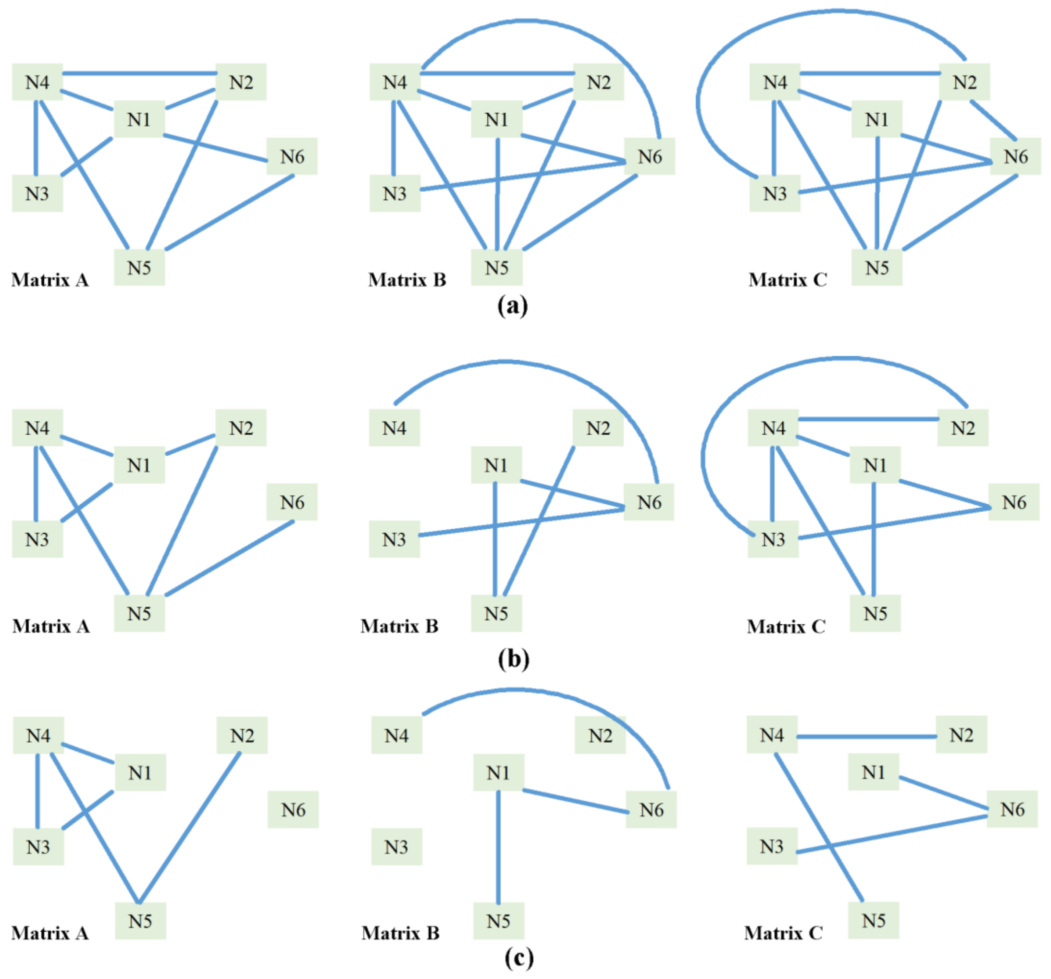

Figure 2 Connectivity graphs in different thresholds. a) Connectivity graphs of matrices A, B and C, are obtained by setting the threshold to be 0.3. b) Connectivity graphs of matrices A, B and C, are obtained by setting the threshold to be 0.5. b) Connectivity graphs of matrices A, B and C, are obtained by setting the threshold to be 0.7.

Threshold setting may have an influence on the final connectivity graphs. Here, a closer analysis on HD and RE are taken in the situations with different thresholds. The real connection of graphs is utilized as the gold standard to evaluate the performance of HD and RE. The lower value in HD and RE, the more similar between two graphs.

Figure 2(a) shows the results with a low threshold (0.3), and B appears to be more similar with A than C. B, and C shares 8 edges. For matrix B, it shares 9 common edges and 2 exceptional edges with matrix A. For matrix C, it shares 8 common edges and 3 exceptional edges with matrix A. HD between matrix A and B is 0.2727, HD between A and C is 0.4545. RE between A and B is 0.6251, RE between A and C is 0.6170. According to the calculated results, differences from RE are too minor to recognize, but HD can recover the fact with an obvious difference.

With an intermediate threshold (0.5) in Figure 2(b), matrix C seems to have minor superiority in similarity than B. For matrix B, it shares 1 common edges and 4 exceptional edges with matrix A. For matrix C, it shares 2 common edges and 6 exceptional edges with matrix A. But totally B and C have the counterpart performance in similarity, this can be validated from calculation results. The HD between A and B is 0.9091, HD between A and C is 0.8182. RE between A and B is 0.9605, RE between A and C is 0.8328.

With a relative high threshold (0.7), HD between matrix A and B is 0.7273, HD between A and C is 0.6364. RE between A and B is 0.8611, RE between A and C is 0.9279. There is little divergence between RE and HD. Here the real connectivity graphs are utilized to evaluate which graph is closer to the standard. As it is shown in Figure 2(c), matrix C has a minor superiority in similarity than B. For matrix B, it shares no common edges and 3 exceptional edges with matrix A. For matrix C, it shares 1 common edges and 3 exceptional edges with matrix A. Thus, HD can always follow the real changing in connection while RE is not that sensitive in distinguish the changes in FCG, despite of the choosing of thresholds in analyzing the FCGs.

RE has its irreplaceable superiority on measuring the difference between graphs, because it can derive an overall evaluation by summing all the differences of the weights on edges. Of course, HD can well evaluate the difference in topographies, but it cannot measure the entire difference on weights. Therefore, two metrics have their unique characteristics, and we should choose the appropriate evaluate metric according to the practical issues.
